# Supplementary material for: Ovarian Cancer in the Era of Precision Surgery and Targeted Therapies
Source: Cancers (Basel). 2025 Oct 18;17(20):3371. doi: 10.3390/cancers17203371 (PMC12562755; doi:10.3390/cancers17203371)
Supplement: Supplementary file 1 [file cancers-17-03371-s001.zip › cancers-3895656-supplementary.pdf]

Table S1. Clinical characteristics of the excluded platinum-sensitive relapsed HGSC patients undergoing cytoreductive relapse surgery

	**Diagnosis (the cohort N=16)**	**Diagnosis (excluded patients N=7)**	**Secondary cytoreductive surgery (the cohort N=16)**	**Secondary cytoreductive surgery (excluded patients N=7)**
Age in years median (range)	64 (41-74)	57 (45-74)	67 (46-77)	60 (47-79)
CA125 median (range)	195 (14-8890)	1000 (38-19400)	35 (6-490)	130 (11-360)
BMI median (range)	24 (18-34)	22 (20-30)	24 (18-35)	23 (19-27)
**Performance score**				
0	9 (56%)	2 (29%)	14 (87.5%)	5 (71%)
1	6 (38%)	2 (29%)	2 (12.5%)	2 (29%)
2	1 (6%)	1 (14%)	-	-
3	-	1 (14%)	-	-
Unknown	-	1 (14%)	-	-
**FIGO stage**				
I-II	4 (25%)	1 (14%)	-	-
III-IV	12 (75%)	6 (86%)	-	-
**Surgery**				
Primary debulking	13 (81%)	1 (14%)	-	-
Interval debulking	3 (19%)	6 (86%)	-	-
**Residual tumor after surgery**				
0	16 (100%)	5 (71%)	14 (87.5%)	5 (71%)
< 1 cm	-	2 (29%)	-	
Unresectable	-	-	2 (12.5%)	2 (29%)
**Platinum response**				
> 12 months (sensitive)	14 (87.5%)	7 (100%)	-	-
6 - ≤ 12 months (partial sensitive)	2 (12.5%)	-	-	-
**Follow-up in months**				
Time to first relapse median (range)	-	-	32 (14-66)	38 (18-104)
Total follow-up time median (range)	-	-	63 (33-122)	63 (32-151)

BMI: Body mass index, CA125: Cancer Antigen 125, EOC: Epithelial ovarian cancer, FIGO: International Federation of Gynecology and Obstretics, HGSC: High grade serous carcinoma.

Table S2. Chemotherapy regimens administered over time

**Diagnosis (N=16)**	
Carboplatin/paclitaxel (6 cycles)	5 (31%)
Carboplatin/docetaxel (6 cycles)	3 (19%)
Carboplatin (6 cycles)	1 (6.25%)
Carboplatin/docetaxel (3+3 cycles)	1 (6.25%)
Carboplatin/paclitaxel (4 cycles) and carboplatin (1 cycle)	1 (6.25%)
Carboplatin (1 cycle) + carboplatin/paclitaxel (5 cycles)	1 (6.25%)
Carboplatin/paclitaxel (3+2 cycles) and carboplatin (1 cycle)	1 (6.25%)
Carboplatin/paclitaxel (2 cycles) and carboplatin (3 cycles)	1 (6.25%)
Carboplatin/paclitaxel (3+4 cycles) and carboplatin (2 cycles)	1 (6.25%)
Carboplatin/paclitaxel (5 cycles) and carboplatin (1 cycle)	1 (6.25%)
**First relapse (N=16)**	
Carboplatin/caelyx (6 cycles)	8 (50%)
Carboplatin (6 cycles)	1 (6.25%)
Carboplatin/caelyx (1 cycle) and carboplatin/gemcitabine (3 cycles)	1 (6.25%)
Carboplatin/caelyx (5 cycles) and carboplatin (1 cycle)	1 (6.25%)
Carboplatin/caelyx (5 cycles)	1 (6.25%)
Carboplatin/caelyx (4 cycles) and caelyx (2 cycles)	1 (6.25%)
Carboplatin (1 cycle)	1 (6.25%)
Carboplatin/caelyx (2 cycles), carboplatin/paclitaxel (2 cycles) and carboplatin (1 cycle)	1 (6.25%)
Carboplatin/paclitaxel (5 cycles)	1 (6.25%)
**Second relapse (N=6)**	
Carboplatin/caelyx (6 cycles)	2 (33.3%)
Carboplatin/docetaxel (3 cycles)	1 (16.7%)
Carboplatin/paclitaxel (5 cycles)	1 (16.7%)
Cisplatin (6 cycles)	1 (16.7%)
No treatment	1 (16.7%)
**Third relapse (N=3)**	
Carboplatin/caelyx (6 cycles)	1 (33.3%)
Carboplatin/gemcitabine (4+4 cycles)	1 (33.3%)
No treatment	1 (33.3%)
**Fourth relapse (N=2)**	
Carboplatin/caelyx (6 cycles)	1 (50%)
No treatment	1 (50%)
**Fifth relapse (N=2)**	
Carboplatin/gemcitabine (3 cycles – still on treatment)	1 (50%)
Paclitaxel (9 cycles)	1 (50%)
**Sixth relapse (N=1)**	
Gemcitabine (3 cycles)	1 (100%)

Table S3. Treatment duration of targeted therapies in months

**Bevacizumab (N=7)**	
Diagnosis (n=3)	13.7, 15.0 and 15.7
First relapse (n=3)	4.4, 15.9* and 20.3*
Second relapse (n=1)	20.1*
**PARP inhibitors (N=10)**	
Diagnosis (n=1)	23.9
First relapse (n=8)	4.2*, 6.9*, 9.7, 12.4*, 14.0, 17.8*, 19.1 and 25.8
Second relapse (n=1)	3.0

PARP: poly(ADP-ribose) polymerase. *Still on treatment at follow-up.

Table S4. Presence of Carcinomatosis at Diagnosis and Relapse

Patient ID	BRCA status	Carcinomatosis at Diagnosis (N=12)	Carcinomatosis at Relapse (N=7)
1	*BRCA2*	Yes	No
3	No	Yes	No
5	*BRCA2*	Yes	No
6	*BRCA1*	Yes	Yes
8	No	Yes	Yes
9	No	No	Yes
10	No	No	Yes
11	No	Yes	No
12	*BRCA2*	Yes	No
13	No	No	Yes
14	No	Yes	Yes
15	No	Yes	No
16	*BRCA2*	Yes	No
17	*BRCA2*	Yes	No
18	No	Yes	Yes
19	No	No	No
